# Ecological Momentary Assessment of Awake Bruxism Frequency in Patients with Different Temporomandibular Disorders

**DOI:** 10.3390/jcm12020501

**Published:** 2023-01-07

**Authors:** Mariana Barbosa Câmara-Souza, Alessandro Bracci, Anna Colonna, Marco Ferrari, Renata Cunha Matheus Rodrigues Garcia, Daniele Manfredini

**Affiliations:** 1 Department of Prosthodontics and Periodontology, Piracicaba Dental School, University of Campinas, Piracicaba 13414-903, Brazil; 2 School of Dentistry, Ingá University Center, Paraná 87035-510, Brazil; 3 Department of Neurosciences, School of Dentistry, University of Padova, 35128 Padova, Italy; 4 Department of Biomedical Technologies, School of Dentistry, University of Siena, 53100 Siena, Italy

**Keywords:** bruxism, temporomandibular joint disorders, ecological momentary assessment, pain

## Abstract

Self-reported awake bruxism (AB) has been associated with temporomandibular disorders (TMD). However, the daily amount of AB behavior has not been quantified in pain patients. Therefore, this study aimed to assess AB frequency in patients with myofascial pain and temporomandibular joint (TMJ) pain and compare it to a group of pain-free individuals. Eighty-four individuals belonging to either a TMD group (*n* = 54) or a healthy control group (*n* = 30) were selected. AB frequency was obtained by ecological momentary assessment with a dedicated smartphone application that sent sound alerts at random intervals during the day for one week. Upon receiving the alert, the volunteer reported the current muscular condition and/or the teeth position, i.e., relaxed jaw muscle, jaw bracing, teeth contact, teeth clenching, or teeth grinding. Data were evaluated by independent *t*-test (α = 0.05). During the seven days, AB frequency was 62.1% ± 26.8% for TMD patients and 36.2% ± 27.3% for pain-free subjects (*p* < 0.001). Mandible bracing was most common in the TMD group (*p* < 0.001), while teeth contact, clenching, and grinding did not differ between groups. Moreover, no differences were found in AB frequency between myofascial pain and TMJ pain patients. Therefore, TMD patients have higher AB frequency characterized by jaw bracing, irrespective of pain location.

## 1. Introduction

Awake bruxism (AB) has been defined as masticatory muscle activity during wakefulness that is characterized by repetitive or sustained tooth contact and/or by bracing or thrusting of the mandible [1]. According to a recent international consensus [1], AB is no longer considered a movement disorder in otherwise healthy individuals. Nonetheless, it may represent a risk factor for negative oral health outcomes, such as mechanical tooth wear, prosthodontic and implant complications, and painful temporomandibular disorder (TMD)-related pain [2]. TMD is an umbrella term that comprises muscle disorders, including myofascial pain with and without mouth-opening limitation, and intra-articular disorders, which encompass disc displacement with or without reduction and mouth-opening limitation, arthralgia, and arthritis [3].

An association between repetitive low-level long-lasting non-functional muscle activity has been reported to cause muscle fatigue and pain [4], and the Orofacial Pain Prospective Evaluation and Risk Assessment (OPPERA) study [5] demonstrated the important role of oral parafunctions on TMD onset. It is important to highlight that TMD is the second most common musculoskeletal disorder that causes pain and disability, demonstrating the relevance of identifying initiating and perpetuating associated factors [6,7]. A previous study using electromyography assessment during standardized tasks showed that TMD patients had approximately 5 to 10 times more AB episodes than pain-free volunteers [8]. However, assessing the presence of waking-state oral behaviors in the natural environment is a challenge due to technical difficulties.

Despite that electromyography-based evaluation has been suggested to achieve a definitive diagnosis of AB [1], a continuous electromyography record for multiple days is uncomfortable for the patient and presents technical difficulties. Therefore, an approach based on Ecological Momentary Assessment (EMA) has been suggested as an alternative option to evaluate AB frequency [9,10]. The EMA approach has been used in the psychological field for some decades [11]; however, it has just recently been introduced for AB assessment, which is also thanks to the use of smartphone-based strategies. With a smartphone application, the frequency of several AB manifestations (i.e., jaw bracing, teeth contact, teeth clenching, and teeth grinding) can be easily monitored over time in a natural environment at multiple daily recording points and for several days [10], by using simple strategies such as sending a sound alert to focus the subject’s attention on the AB conditions [12].

Until now, EMA has been used to investigate the frequency of AB in healthy young adults—specifically university students [10,13,14,15,16,17], pre-college students [18], and patients undergoing orthodontic treatment [19]—and only a single study has evaluated it on the general population [20]. These studies elucidated the report of AB in otherwise healthy young adults and are considered standpoints for future comparison with selected populations of patients with purported bruxism risk factors and/or consequences [1]. Conversely, no previous study has assessed the different manifestations of AB in a TMD population by using a dedicated application. The literature has only focused on teeth clenching and teeth contacting habits [21,22] without considering sustained muscle activity without teeth contact (i.e., jaw bracing) [1,21,22].

Therefore, information about the AB characteristics in TMD patients is limited, especially considering a comparison with healthy individuals, which may improve knowledge on AB as a risk factor for TMD and further clarify some possible pathophysiological mechanisms that are implicated in symptom onset [23]. Thus, this study aimed to assess the frequency of AB conditions in patients with myofascial pain and temporomandibular joint (TMJ) pain and to compare these patients to a control group of pain-free individuals.

## 2. Materials and Methods

### 2.1. Experimental Design

This cross-sectional study investigated AB frequency in TMD patients and pain-free volunteers. Participants in the TMD group were selected from consecutive patients attending the Orofacial Pain Clinic at the University of Siena, Italy. Participants in the control group were selected from consecutive patients attending the School of Dentistry at the University of Siena, Italy, for conservative dental care. Volunteer selection occurred without gender or ethical restrictions.

To be included in the study, volunteers needed to have good general health, received at least one painful diagnosis according to the diagnostic criteria for temporomandibular disorders (DC/TMD) [3], and owned a smartphone compatible with the application being used. Participants with psychiatric, neurological, or uncontrolled systemic diseases and those with previous head and/or neck trauma were excluded. To be included in the control group, participants had to follow the same inclusion and exclusion criteria except that they should be free of any orofacial pain and not have any TMD diagnosis.

All volunteers invited to participate had to sign a consent form, which was approved by the local IRB (#344-CES-AULSS9) and followed the Helsinki Declaration. Participants were assured that they could withdraw from the study at any time without any nuisance.

### 2.2. AB Condition Assessment

AB frequency was obtained using the EMA approach by use of the BruxApp^®^ smartphone application (WMA srl) to assess five oral conditions. The application is programmed to send 20 sound alerts at random intervals during the day, with the aim of reducing expectation bias. The subject can choose the type of sound alert to distinguish it from other notifications. Recording time was set from 8:00 to 12:30 and from 14:30 to 22:00 to allow a break during lunchtime [10].

Before starting the evaluation period, the investigator provided detailed information on how to use the smartphone application and how to identify each condition, since it has been proved that training participants on such manifestations provides better understanding and compliance [24]. The conditions were described as follows [10]:

- Relaxed jaw muscles: condition of perceived jaw muscles relaxation, with mandibles kept apart;
- Teeth contact: condition of slight teeth contact similar to the teeth contact that the subject perceives when a 40 μ articulating paper (Bausch Occlusions papier^®^; Bausch KG, Koln, Germany) is put between the dental arches and he/she is asked to slightly keep the teeth in contact to retain it on site. In short, this condition is defined as a light touching of teeth when the mouth is closed;
- Teeth clenching: all conditions in which teeth contact is more marked than the above and jaw muscles are kept tense;
- Jaw clenching (without teeth contact): condition of jaw muscle stiffness or tension similar to teeth clenching but with teeth kept apart (i.e., bracing);
- Teeth grinding: a condition in which the opposite teeth are gnashed or ground independent of intensity and direction of antagonist teeth contact.

For a better understanding of how to recognize the five behaviors, an educational video recorded by the project coordinator (DM) could be accessed by the participants (https://www.youtube.com/watch?v=xL79AcnpBCY&t=15s).

When receiving the alert, the participant had to focus attention on the mouth area and tap on the display icon corresponding to the current muscular condition and/or teeth position. A time span of up to 5 min within the alert sound was allowed. If the participant tried to reply after five minutes, the answer was not stored in the software (unusable data). Additionally, alerts received during functional activities, such as eating or talking, were to be discarded. Data recording was followed for 7 days, and a minimum of 60% replies was necessary to consider the day as valid (i.e., a minimum of 12 alerts answered per day out of 20 alerts programmed). In case of failure to reach the minimum percentage of valid answers, the app automatically generated one additional day to complete the 7 valid day protocol. When completing the observation period, an anonymous file was generated (.csv format), and the participant was instructed to send it to the researchers via dedicated email.

### 2.3. Statistical Analysis

The collected data were evaluated by means and frequencies of each AB condition. Data showed normal distribution as verified by the Shapiro–Wilk test. Then, between-group and between-gender comparisons were performed using independent *t*-test. Moreover, TMD patients were also compared according to the presence of joint or myofascial pain symptoms. All statistical analyses were performed using the SPSS 21.0 (IBM Corp. Released 2012. IBM SPSS Statistics for Windows, Version 21.0. Armonk, NY, USA: IBM Corp.) considering a significance level of 5%.

## 3. Results

One hundred five individuals were invited to participate in this study. However, 21 of them were excluded due to lack of compliance in replying to the alerts. Thus, 84 participants comprised the final sample: 54 of them belonging to the TMD group (46 females; mean age = 36.6 ± 11.1) and 30 non-TMD volunteers as the control group (17 females; mean age = 41.0 ± 10.1).

The smartphone app should have been used for one week; however, several participants did not reply to at least 60% of the alerts over seven consecutive days. The mean compliance recorded with the smartphone application was 70.4 ± 8.1% (range 61.4–92.9%) of the total alerts. On average, 9.3 ± 2.9 days (range 7–16) were necessary to achieve the targeted goal of 7 days with a minimum of 60% alerts/day. No gender differences were detected in any compliance data.

Potential gender-related differences in AB values were investigated. All AB manifestations, except for bracing, did not show statistically significant differences between males and females (*p* > 0.05). Considering bracing behavior, females had a frequency almost twice as high as males (26.6% ± 25.1% vs. 14.0% ± 14.6%, respectively; *p* = 0.007).

During the 7 valid days, AB frequency was 62.1% ± 26.8% for TMD patients and 36.2% ± 27.3% for pain-free subjects (*p* < 0.001). Detailed values for each of the AB manifestations according to each group can be found in Table 1.

In addition, considering that TMD is an umbrella term comprising various muscle and joint disorders, the frequency of AB behavior was also assessed according to each manifestation. However, no differences were found between those experiencing myofascial pain and those with joint disorders (Table 2).

## 4. Discussion

The present study used a dedicated smartphone application to assess the frequency of four different AB manifestations. It was possible to observe that patients with TMD-related pain had a significantly higher frequency of AB when compared to pain-free adults. It was interesting to notice that significant differences were achieved for the jaw bracing activity, while teeth contact, clenching, and grinding were similar between groups, with a very low frequency of grinding in both groups.

The concept of mandible bracing was emphasized in the 2018 international consensus, in which AB was definitively characterized as a muscle activity, irrespective of teeth contact [1]. This represents an important change concerning past views of bruxism as a teeth-induced disorder [25,26]. Thus, understanding the concept and recognition of jaw bracing is an important step to deal with AB, which could lead to relevant clinical consequences. Jaw bracing is likely characterized by sustained low-level long-lasting muscle contraction, which is almost imperceptible to the patient, being a voluntary but unconscious activity. This prolonged contraction is likely to cause muscle stiffness and TMJ overload, which justifies this higher frequency in TMD patients.

Additionally, it must be highlighted that none of the teeth-involving manifestations (i.e., teeth contact, teeth clenching, and teeth grinding) were different between groups. Although previous reports had not examined the jaw bracing condition, some authors [4] found that teeth contact and clenching were significantly associated with orofacial pain. Previous studies have demonstrated that almost 50% of TMD-pain patients keep teeth together during wakefulness [27], and that they have four times more non-functional dental contacts than healthy individuals [21]. Further, teeth contact prevalence, even measured by self-reported data, was higher than teeth clenching in participants with and without pain [4]. This is in accordance with the present findings, which report that tooth contact had a mean frequency of around 24%, while teeth clenching had a frequency of 8%.

Considering former studies using the same app to collect data on AB behaviors, the healthy subjects included in this research had a similar AB frequency over the seven evaluation days. Although sample characteristics are different among the investigations on young adults [10,12,13,14,15,16,17,18], the daily stressful situations of the modern era may lead to the similar frequency of AB reported in all asymptomatic individuals. Further, the only previous study comprising the general population [20] also found similar results for AB frequency in healthy participants, suggesting that a certain amount of AB behaviors is physiological in otherwise healthy people [1].

Interestingly, within the TMD patients, no differences in AB frequency were found between patients with muscle vs. joint pain. This finding suggests that the onset and location of symptoms are likely host-mediated and that a common physiopathology of muscle-induced overload may exist for muscle and joint symptoms. This hypothesis is also in line with previous studies reporting that AB is a risk factor for the presence of articular pain in patients with TMJ clicking as well as for intermittent locking [28,29].

Some limitations should be remarked. Regarding participants’ compliance, the present study lost approximately 25% of the respondents during the week-long interval, highlighting slightly lower compliance compared to the reference study, and pointing out the need to address this issue as part of any future study protocol [30]. Nonetheless, some minor differences in compliance rates between studies may just be due to the different age groups that are involved in the investigations, with youngsters being more sensitive to the use of smartphones. Concerning the study groups, it was not possible to match them by gender, since the TMD group that completed the observation period had a higher proportion of females than the control group. Nonetheless, the literature suggests that AB prevalence does not differ between genders [22], which was confirmed by our findings, thus suggesting that group composition did not influence our results. In addition, although smartphone-based EMA is a potentially reliable instrument to report wake-state oral behavior in the natural environment, the fact that it is still based on self-reporting cannot be underestimated [1]. However, collecting real-time data at multiple points throughout the day recalls the patient’s attention very close in time to the event (alerts replied to after 5 min are not considered) and can be considered a suitable strategy to assess AB behaviors and improve studies on the topic [10]. Moreover, to clearly understand the different manifestations of such activities, a population-representative sample size is indicated. It would also be relevant to collect data on stress sensitivity during the evaluation period, which could clarify the hypotheses related to AB etiology [31]. Finally, considering the psychosocial aspects involved in AB and TMD-related pain, assessing such outcomes would be extremely relevant to elucidate possible associated factors [32,33].

## 5. Conclusions

TMD patients have higher AB frequency than healthy individuals, and such manifestations are mainly characterized by jaw bracing. Thus, managing AB could be an important step to reduce pain symptoms, and instructing patients regarding physiological muscle positioning could prevent TMD onset.

## Figures and Tables

**Table 1:** Mean (SD) frequencies of awake bruxism behavior of participants with and without temporomandibular disorders.

	Non-TMD (*n* = 30)	TMD (*n* = 54)	*p*-Value
Relaxed	63.8 (27.4)	37.8 (26.4)	<0.001
Jaw Bracing	12.7 (17.4)	29.4 (24.5)	<0.001
Teeth Contact	18.4 (20.1)	24.0 (21.5)	0.248
Teeth Clenching	4.0 (10.6)	8.0 (12.9)	0.151
Teeth Grinding	1.0 (3.9)	0.8 (3.4)	0.729
Total AB frequency	36.3 (27.5)	62.2 (26.6)	<0.001

TMD, temporomandibular disorders; AB, awake bruxism; SD, standard deviation.

**Table 2:** Mean (SD) values of each AB manifestation considering muscle TMD only or joint TMD associated with/without muscle pain.

	TMD Diagnosis		*p*-Value *
AB Manifestation	Myofascial Pain (*n* = 26)	TMJ Pain (*n* = 28)	
Bracing	27.3 (25.8)	31.4 (23.4)	0.543
Teeth Contact	24.0 (21.5)	24.0 (21.9)	0.982
Teeth Clenching	10.0 (15.0)	6.2 (10.4)	0.290
Teeth Grinding	1.1 (4.8)	0.5 (1.3)	0.496
Total AB frequency	62.4 (29.7)	62.0 (24.3)	0.956

* Independent *t*-test.

## Data Availability

Data will be made available by the authors upon request.

## References

[1] Lobbezoo F., Ahlberg J., Raphael K.G., Wetselaar P., Glaros A., Kato T., Santiago V., Winocur E., De Laat A., De Leeuw R. (2018). International consensus on the assessment of bruxism: Report of a work in progress. J. Oral Rehabil..

[2] John M.T., Reissmann D.R., Schierz O., Wassell R.W. (2007). Oral health-related quality of life in patients with temporomandibular disorders. J. Orofac. Pain.

[3] Schiffman E., Ohrbach R., Truelove E., Look J., Anderson G., Goulet J.-P., List T., Svensson P., Gonzalez Y., Lobbezoo F. (2014). Diagnostic criteria for temporomandibular disorders (DC/TMD) for clinical and research applications: Recommendations of the International RDC/TMD Consortium Network* and Orofacial Pain Special Interest Group. J. Oral Facial Pain Headache.

[4] Glaros A.G., Williams K. (2012). Tooth contact versus clenching: Oral parafunctions and facial pain. J. Orofac. Pain.

[5] Ohrbach R., Bair E., Fillingim R.B., Gonzalez Y., Gordon S.M., Lim P.F., Ribeiro-Dasilva M., Diatchenko L., Dubner R., Greenspan J.D. (2013). Clinical orofacial characteristics associated with risk of first-onset TMD: The OPPERA prospective cohort study. J. Pain.

[6] Valesan L.F., Da-Cas C.D., Réus J.C., Denardin A.C.S., Garanhani R.R., Bonotto D., Januzzi E., de Souza B.D.M. (2021). Prevalence of temporomandibular joint disorders: A systematic review and meta-analysis. Clin. Oral Investig..

[7] Jin L.J., Lamster I.B., Greenspan J.S., Pitts N.B., Scully C., Warnakulasuriya S. (2016). Global burden of oral diseases: Emerging concepts, management and interplay with systemic health. Oral Dis..

[8] Cioffi I., Landino D., Donnarumma V., Castroflorio T., Lobbezoo F., Michelotti A. (2017). Frequency of daytime tooth clenching episodes in individuals affected by masticatory muscle pain and pain-free controls during standardized ability tasks. Clin. Oral Investig..

[9] Manfredini D., Bracci A., Djukic G. (2016). BruxApp: The ecological momentary assessment of awake bruxism. Minerva Stomatol..

[10] Bracci A., Djukic G., Favero L., Salmaso L., Guarda-Nardini L., Manfredini D. (2018). Frequency of awake bruxism behaviours in the natural environment. A 7-day, multiple-point observation of real-time report in healthy young adults. J. Oral Rehabil..

[11] Shiffman S., Stone A.A. (1998). Ecological momentary assessment in health psychology. Health Psychol..

[12] Bracci A., Lobbezoo F., Häggman-Henrikson B., Colonna A., Nykänen L., Pollis M., Ahlberg J., Manfredini D., International Network for Orofacial Pain and Related Disorders Methodology INfORM (2022). Current Knowledge and Future Perspectives on Awake Bruxism Assessment: Expert Consensus Recommendations. J. Clin. Med..

[13] Zani A., Lobbezoo F., Bracci A., Ahlberg J., Manfredini D. (2019). Ecological momentary assessment and intervention principles for the study of awake bruxism behaviors, Part 1: General principles and preliminary data on healthy young Italian adults. Front. Neurol..

[14] Dias R., Vaz R., Rodrigues M.J., Serra-Negra J.M., Bracci A., Manfredini D. (2021). Utility of Smartphone-based real-time report (Ecological Momentary Assessment) in the assessment and monitoring of awake bruxism: A multiple-week interval study in a Portuguese population of university students. J. Oral Rehabil..

[15] Emodi-Perlman A., Manfredini D., Shalev T., Yevdayev I., Frideman-Rubin P., Bracci A., Arnias-Winocur O., Eli I. (2021). Awake Bruxism-Single-Point Self-Report versus Ecological Momentary Assessment. J. Clin. Med..

[16] Zani A., Lobbezoo F., Bracci A., Djukic G., Guarda-Nardini L., Favero R., Ferrari M., Aarab G., Manfredini D. (2021). Smartphonebased evaluation of awake bruxism behaviours in a sample of healthy young adults: Findings from two University centres. J. Oral Rehabil..

[17] Emodi-Perlman A., Manfredini D., Shalev T., Bracci A., Frideman-Rubin P., Eli I. (2021). Psychosocial and Behavioral Factors in Awake Bruxism-Self-Report versus Ecological Momentary Assessment. J. Clin. Med..

[18] Câmara-Souza M.B., Carvalho A.G., Figueredo O.M.C., Bracci A., Manfredini D., Rodrigues Garcia R.C.M. (2020). Awake bruxism frequency and psychosocial factors in college preparatory students. Cranio.

[19] Pereira N.C., Oltramari P.V.P., Conti P.C.R., Bonjardim L.R., de Almeida-Pedrin R.R., Fernandes T.M.F., de Almeida M.R., Conti A.C.C.F. (2021). Frequency of awake bruxism behaviour in orthodontic patients: Randomised clinical trial: Awake bruxism behaviour in orthodontic patients. J. Oral Rehabil..

[20] Bucci R., Manfredini D., Lenci F., Simeon V., Bracci A., Michelotti A. (2022). Comparison between Ecological Momentary Assessment and Questionnaire for the Assessment of Frequency of Waking-Time Non-Functional Oral Behaviours. J. Clin. Med..

[21] Funato M., Ono Y., Baba K., Kudo Y. (2014). Evaluation of the non-functional tooth contact in patients with temporomandibular disorders by using newly developed electronic system. J. Oral Rehabil..

[22] Chen C.Y., Palla S., Erni S., Sieber M., Gallo L.M. (2007). Nonfunctional tooth contact in healthy controls and patients with myogenous facial pain. J. Orofac. Pain.

[23] Manfredini D., De Laat A., Winocur E., Ahlberg J. (2016). Why not stop looking at bruxism as a black/white condition? Etiology could be unrelated to clinical consequences. J. Oral Rehabil..

[24] Nykänen L., Manfredini D., Lobbezoo F., Kämppi A., Colonna A., Zani A., Almeida A.M., Emodi-Perlman A., Savolainen A., Bracci A. (2022). Ecological Momentary Assessment of Awake Bruxism with a Smartphone Application Requires Prior Patient Instruction for Enhanced Terminology Comprehension: A Multi-Center Study. J. Clin. Med..

[25] Manfredini D., Winocur E., Guarda-Nardini L., Paesani D., Lobbezoo F. (2013). Epidemiology of bruxism in adults: A systematic review of the literature. J. Orofac. Pain.

[26] Lobbezoo F., Ahlberg J., Manfredini D., Winocur E. (2012). Are bruxism and bite causally related?. J. Oral Rehabil..

[27] Sato F., Kino K., Sugisaki M., Haketa T., Amemori Y., Ishikawa T., Shibuya T., Amagasa T., Shibuya T., Tanabe H. (2006). Teeth contacting habit as a contributing factor to chronic pain in patients with temporomandibular disorders. J. Med. Dent. Sci..

[28] Poluha R.L., De la Torre Canales G., Bonjardim L.R., Conti P.C.R. (2021). Clinical variables associated with the presence of articular pain in patients with temporomandibular joint clicking. Clin. Oral Investig..

[29] Kalaykova S., Lobbezoo F., Naeije M. (2011). Risk factors for anterior disc displacement with reduction and intermittent locking in adolescents. J. Orofac. Pain.

[30] Colonna A., Lombardo L., Siciliani G., Bracci A., Guarda-Nardini L., Djukic G., Manfredini D. (2020). Smartphone-based application for EMA assessment of awake bruxism: Compliance evaluation in a sample of healthy young adults. Clin. Oral Investig..

[31] Manfredini D., Landi N., Fantoni F., Segù M., Bosco M. (2005). Anxiety symptoms in clinically diagnosed bruxers. J. Oral Rehabil..

[32] Manfredini D., Lobbezoo F. (2009). Role of psychosocial factors in the etiology of bruxism. J. Orofac. Pain.

[33] Colonna A., Guarda-Nardini L., Ferrari M., Manfredini D. (2021). COVID-19 pandemic and the psyche, bruxism, temporomandibular disorders triangle. Cranio.

